# Diverging views between clinicians, service users, family caregivers and researchers on the classification of restrictive practices in mental health services

**DOI:** 10.1017/S2045796025100322

**Published:** 2025-12-12

**Authors:** Zelalem Belayneh, Den-Ching A. Lee, Melissa Petrakis, Deborah Aluh, Justus Uchenna Onu, Giles Newton-Howes, Masters Kim, Yoav Kohn, Jacqueline Sin, Marie-Hélène Goulet, Tonje Lossius Husum, Eleni Jelastopulu, Maria Bakola, Sau Fong Leung, Kathleen De Cuyper, Eimear Muir-Cochrane, Yana Canteloupe, Emer Diviney, Lesley Barr, Jim Ridley, Didier Demassosso, Terry P. Haines

**Affiliations:** 1 School of Primary and Allied Health Care, Faculty of Medicine, Nursing and Health Sciences, Monash University, Melbourne, Australia; 2 Department of Psychiatry, Dilla University, Dilla, Ethiopia; 3 Rehabilitation, Ageing and Independent Living Research Centre, Monash University, Melbourne, Victoria, Australia; 4 National Centre for Healthy Ageing, Peninsula Health and Monash University, Melbourne, Victoria, Australia; 5 Mental Health Services, St Vincent’s Hospital, Melbourne, Australia; 6 Lisbon Institute of Global Mental Health, Comprehensive Health Research Centre (CHRC), NOVA Medical School, NOVA University of Lisbon, Lisbon, Portugal; 7 Department of Clinical Pharmacy, University of Nigeria Nsukka, Nsukka, Nigeria; 8 Department of Mental Health, Nnamdi Azikiwe University, Awka, Nigeria; 9 Department of Training and Research, Federal Neuropsychiatric Hospital, Enugu, Nigeria; 10 School of Philological Medicine, University of Otago, Wellington, New Zealand; 11 College of Health Professions, Department of Clinical Sciences, Division of Physician Assistant Studies, Medical University of South Carolina, Charleston, SC, USA; 12 School of Medicine, Jerusalem Mental Health Centre, Hebrew University-Hadassah, Jerusalem, Israel; 13 School of Health and Medical Sciences, City St George’s, University of London, London, UK; 14 Faculty of Nursing, Université de Montréal, Québec, Canada; 15 Centre de recherche de l’ Institut universitaire en santé mentale de Montréal, Québec, Canada; 16 Faculty of Health Sciences, Oslo Metropolitan University, Oslo, Norway; 17 Department of Public Health, Epidemiology and Quality of Life, School of Medicine, University of Patras, Patras, Greece; 18 School of Nursing, The Hong Kong Polytechnic University, Hong Kong, China; 19 LUCAS – Center for Care Research & Consultancy, KU Leuven, Belgium; 20 College of Nursing and Health Sciences, Flinders University, Adelaide, South Australia; 21 Adult Community and Rehabilitation Stream, Eastern Health, Melbourne, Victoria, Australia; 22 Self Help Addiction Resource Centre, Melbourne, Victoria, Australia; 23 Curtin School of Nursing, Faculty of Health Sciences, Curtin University, Perth, Australia; 24 Nursing and Governance, Greater Manchester Mental Health Trust, Prestwich, UK; 25 Green Ribbon Health and Community Development Association (GriCoDa), Yaounde, Cameroon

**Keywords:** classification, coercion, interpretation, perspective, restraint, restrictive practices, seclusion, sedation, stakeholder perspectives, stakeholders

## Abstract

**Aims:**

Efforts to reduce restrictive practices (RPs) in mental health care are growing internationally. Yet, inconsistent definitions and perspectives often challenge the consistent implementation and evaluation of reduction strategies. This study explored which scenarios different mental health stakeholders classify as RPs, examined the contextual factors influencing these classifications and compared classification patterns across clinicians, researchers, service users and family caregivers.

**Methods:**

An international cross-sectional survey was conducted using a multilingual online questionnaire hosted on the Qualtrics platform. A total of 851 stakeholders participated, including clinicians (*n* = 517), service users (*n* = 80), family caregivers (*n* = 89) and researchers (*n* = 165). Participants were presented with 44 potential RP case scenarios and asked to rate whether each scenario should be classified as an RP using a four-point Likert scale (Definitely yes, Probably yes, Probably no, Definitely no). The scenarios were organized into 22 paired comparisons, each sharing the same core context but differing in specific details. Paired comparisons were analyzed one pair at a time, allowing us to identify classification patterns between the scenarios and isolate the effects of particular contextual factors using ordered logistic regression. Interaction analyses were then conducted to assess how classification patterns varied across stakeholder groups.

**Results:**

Substantial discrepancies exist both within and between stakeholder groups regarding whether a given action should be considered an RP or not. Physically visible actions were often identified as RPs across all groups, while less visible forms often went unrecognized. Contextual differences, such as the healthcare professional’s intention, duration of the action, methods used, presence or absence of consent, door-locking status, and the severity of anticipated harm to be prevented influenced whether a given action was classified as an RP. Service users classified more scenarios as RPs than other groups; however, their decisions were more context-sensitive, shifting notably even with minor changes in scenario details. Among the 22 paired scenarios compared, 13 (59.09%) showed significant differences (*p* < 0.01) within at least one stakeholder group and eight demonstrated differences between groups.

**Conclusions:**

Mental health stakeholders’ interpretations of RPs were often shaped not only by the inherent coercive nature of actions but also by the context in which they occurred and the professional role of the assessors. This underscores the need for harmonized definitions and classification frameworks for RPs, co-designed with diverse stakeholders. Addressing less visible forms of RPs in policy and clinical practice is also essential.

## Introduction

In mental health settings, various restrictive practices (RPs) such as physical or mechanical restraint, seclusion, chemical restraint, compulsory admission and informal coercion are commonly employed in response to behaviours perceived as posing a risk of harm to oneself or others (World Health Organization, 2019). RPs are broadly defined as making someone do something they don’t want to do or stopping someone from doing something they want to do (Britain, 2014). The use of RPs limits the dignity, autonomy, and freedom of movement of individuals and has the potential to cause serious physical and psychological harm (Chieze *et al.*, 2019).

Reducing RPs has been one of the forefront issues in international mental health reform over the past two decades. They are considered to be used only as a last resort (Power *et al.*, 2020). A range of policy reforms and evidence-based frameworks has been introduced to support this goal, including the Open Wards Policy (Jungfer *et al.*, 2014), the Six Core Strategies (Riahi *et al.*, 2016), the Safewards model (Maguire *et al.*, 2018) and trauma-informed care (Forkey *et al.*, 2021). A key strategy across these frameworks is the integration of reduction strategies into routine clinical care and the replacement of RPs with less restrictive alternatives (Muluneh *et al.*, 2025).

The effective translation of these principles into practice requires meaningful engagement with a diverse range of mental health stakeholders, including service users and family caregivers (Bennetts *et al.*, 2024). However, inconsistent definitions of RPs (Muluneh *et al.*, 2025) and differing stakeholder perspectives hinder shared decision-making, complicating both the practical implementation of policy reforms and the successful reduction of RPs. Healthcare professionals often view RPs as necessary for safety and ward management, whereas many service users often perceive them as harmful and as violations of human rights (Mayers *et al.*, 2010). Whether or not specific actions are interpreted as RPs is highly influenced by the divergent perspectives of various stakeholders. This challenges the ability to reach a consensus on what constitutes acceptable routine clinical interventions versus RPs that should be avoided (Murphy *et al.*, 2021). Thus, inconsistencies in stakeholder perspectives may be a powerful yet under-recognized factor perpetuating unnecessary reliance on RPs, even in systems formally committed to their reduction.

Several factors including cultural perspectives, legal frameworks, professional training, individual values, shared assumptions, epistemic disparities and lived experiences can affect individuals’ views towards RPs (McSherry, 2013). Additionally, variations in the context in which an action occurs can lead to different interpretations of the same practice. Factors such as the presence or absence of consent, the healthcare professional’s intent, the degree of restriction, the duration of the action, whether doors are locked, whether staff or security initiated the intervention, and whether the action is actually implemented or merely threatened are all likely to influence its classification (Duffy *et al.*, 2023). Despite this, few studies have systematically examined how contextual factors affect RP classification across diverse stakeholder groups or explored differences in perspectives for various types of RPs.

This study sought to address two aims related to how different mental health stakeholders conceptualize and classify RPs. First, it sought to characterize the types of scenarios that stakeholders consider should be classified as RPs. Second, it sought to identify the contextual factors that were influential in determining whether a scenario should be considered an RP and to compare these factors between stakeholder groups.

## Methods

### Design and participants

This study was an international cross-sectional survey involving clinicians, service users, caregivers and researchers. Eligibility criteria varied by participant group. Clinicians included nurses, psychiatrists, psychologists, social workers, medical practitioners, occupational therapists and other trained staff currently working in adult mental health inpatient settings. Service users were individuals with lived experience in such settings and having been discharged six months prior to data collection. Family caregivers were individuals who provided unpaid support to a relative, friend or significant other admitted to an adult mental health inpatient facility. Individuals under 18 years of age or those receiving or providing inpatient care during the survey administration were excluded. The researchers’ group included those who self-identified as researchers with expertise in mental health inpatient care and/or RPs.

### Development of survey strategies and instruments

The development of the overall instruments and data collection strategies for this survey followed a co-designed approach, informed by a series of systematic reviews (Belayneh *et al.*, 2024, 2025b; Muluneh *et al.*, 2025). The co-design process involved 33 mental health stakeholders, including clinicians, service users, caregivers, and researchers, representing 17 countries across Africa, Asia, North America, Europe, and Australia (Belayneh *et al.*, 2025a). A detailed methodological description of the survey is available in the published protocol (Belayneh *et al.*, 2025b).

**Figure 1.**
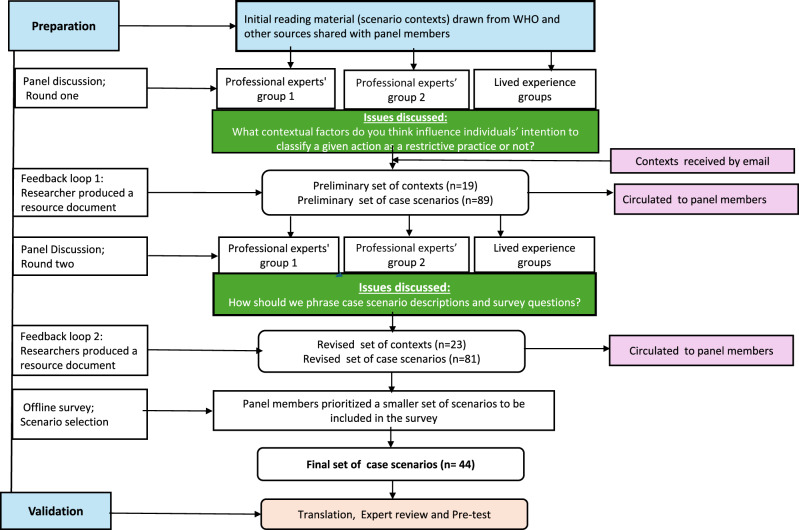
Structure and overall step-by-step process of the co-design work.

Two rounds of online panel meetings were held (Figure 1). The first round aimed to explore contextual elements influencing how individuals classify an action as a RP, resulting in the identification of 23 key contextual elements that guided the development of case scenario descriptions. These included whether a door was locked or unlocked, the duration of the intervention, the identity of the person administering it, the presence or absence of a risk assessment, attempts at less restrictive alternatives, legal approval, and documented evidence prompting the intervention.

In the second round, panel members drafted 81 case scenarios reflecting various contexts to illustrate real-world situations (Supplementary File 1). From these, 44 scenarios were prioritized for survey data collection, covering a range of RPs: 21 mechanical restraint, 8 manual restraint, 16 chemical restraint, 17 seclusion, 4 compulsory admission, 4 informal coercion and 11 involving combined methods. The full list of scenario descriptions is available in Supplementary File 2. Several comparator scenarios were developed within each context, featuring identical core descriptions but differing by a single situational element (see underlined statements in the examples below). For instance, in the door-locking context, two comparator scenarios differ only in which door is locked:

1. A nurse locks the individual person’s room door while the person is inside to prevent escape from the hospital.
2. A nurse locks the main ward door, leaving the individual person’s room door open to prevent escape from the hospital.

This design was chosen to isolate and assess the influence of specific contextual elements on the participants’ decisions regarding whether or not an action constituted an RP.

### Recruitment and outcome measurement

Participants were recruited online using the Qualtrics platform, enabling respondents from any country worldwide. Qualtrics is a widely used survey tool offering secure data collection, customizable question formats, and automated data management (Carter and Ponte, 2022). Its user-friendly interface, multi-device compatibility, and robust features for handling complex survey logic ensured accurate and efficient data collection for this international study (Boas *et al.*, 2020; Molnar, 2019). An anonymous survey link and a flyer with a QR code were widely distributed through institutional websites and social media channels, including X, Facebook, Instagram and LinkedIn. Various institutions, professional associations and mental health advocacy groups supported recruitment by hosting the survey, sharing it via mailing lists, and featuring it in newsletters. Additionally, a snowball sampling technique was employed, with international research team members actively promoting the survey through their professional and personal networks and acting as regional contacts for data collection.

The survey questionnaire was professionally translated and expert-reviewed in English, Amharic, Dutch, French, Hebrew, and Greek, allowing participants to select their preferred language via the multilingual survey link.

Outcome data were collected by inviting survey respondents to review narrative descriptions of 44 case scenarios and respond to a closed-ended question asking whether they believed the action depicted constituted an RP. Responses were recorded using a four-point Likert scale: Definitely yes, Probably yes, Probably no or Definitely no.

### Data analysis

Descriptive statistics, including frequencies and percentages, summarized participant characteristics and the proportion of participants who classified each scenario as a restrictive practice or not. The study employed ordered logistic regression to examine whether the classification of scenarios as RPs was influenced by differences in the specific contextual categories embedded within each scenario, and to identify which contextual factors contributed to discrepancies in scenario classification both within and between stakeholder groups. These models accounted for the ordinal nature of the 4-point Likert scale responses (‘Definitely yes’, ‘Probably yes’, ‘Probably no’ and ‘Definitely no’). The model was adjusted for respondent ID, with clustered robust standard errors applied to account for repeated measures within participants.

Separate regression analyses were conducted for responses received from clinicians, researchers, service users, and family caregivers to examine within-group differences in scenario classification. A pairwise comparison approach was also utilized, in which each pair of comparator scenarios shared an identical core contextual description but differed in one specific situational detail. A total of 22 paired comparisons were conducted to examine classification patterns both within and across stakeholder groups. For example, in the context of handholding, two scenarios were compared: one involved a nurse firmly holding a person’s arms to prevent self-harm, while the other involved a security staff member performing the same action. Additionally, interaction effect analyses were performed to determine whether specific contextual elements of the scenarios had varying effects across stakeholder groups (clinicians, researchers, service users and family caregivers). Comparisons of other stakeholders’ classifications were made with reference to clinicians’ classifications, given the larger number of clinicians who participated in the study, which can maximize the statistical power of interaction analyses. Additionally, clinicians are the stakeholder group primarily responsible for recording instances of RPs (Grace-Martin, 2020). This approach allowed us to examine how clinicians’ perspectives align with or differ from those of other stakeholders. These models revealed four patterns of scenarios: (1) consistent differences in classification across all stakeholder groups, (2) variation specific to certain groups but not others, (3) no significant effect in any stakeholder group and (4) significant variation within stakeholder groups, but not between them.

All statistical analyses were performed using STATA version 17, with statistical significance set at *p* < 0.05 and a 95% confidence level.

## Results

### Characteristics of study participants

A total of 864 participants from 47 countries completed the core set of scenario items in this study. The regional representation includes Africa (39.26%), Europe (34.18%), Asia (8.59%), North America (3.52%) and Australasia (14.71%). The sample included 517 clinicians, 165 researchers, 80 service users and 89 family caregivers.

### Classification of RP scenarios

Participants’ responses varied considerably in how they classified the same potential RP scenarios, both between and within stakeholder groups. The service user group more frequently classified scenarios as RPs, providing the highest number of ‘Definitely yes’ responses for 42 out of 44 scenarios (95.45%) compared to classifications made by clinicians, family caregivers and researchers. In contrast, family caregivers recorded the lowest proportion of ‘Definitely yes’ responses for 31 out of 44 scenarios (70.45%).

Overall, the proportion of ‘Definitely yes’ responses for classifying each scenario as an RP was relatively low across stakeholder groups. The highest proportions of ‘Definitely yes’ responses for specific scenarios were as follows: 86.44% among service users for Scenario 22 (restraint without prior risk assessment), 72.38% among clinicians and 66.00% among family caregivers for Scenario 27 (mechanical restraint of both wrists and ankles), and 60.9% among researchers for Scenario 14 (restraint without consent).

Detailed data on the frequency and proportion of participants’ responses for the classification of each of the 44 case scenarios, indicating whether or not they were identified as RPs are provided in Supplementary File 2. The data are presented as cross-tabulations comparing responses across stakeholder groups.

### Scenario contexts influencing scenario classification within each stakeholder group

Nine of the 22 contextual elements of RP examined by our comparative scenarios did not influence any of the stakeholder groups’ responses regarding whether the scenarios described were an RP or not (Tables 1 and 2). These elements included: the time of day when the action took place (daytime or nighttime), type of shift (morning or afternoon), whether less restrictive alternatives were attempted before the action, the type of activities the person was prevented from engaging in during the episode (e.g., receiving visitors or leaving designated areas), and the availability of utilities in the seclusion room (empty or furnished). These contexts therefore appear to be less important for inclusion in future work seeking to improve the classification and recording of RPs.

**Table 1.** Comparison of the influence of variations in specific contextual factors/definitional elements on scenario classification where or not as restricitve practice among individuals within each stakeholder group

		Participant groups			
		Clinicians	Researchers	Service users	Family caregivers
		Odds ratio (95% CI), *p*-value			
Contextual factors/definitional element of scenarios compared	Scenarios contrasted	(OR > 1 indicate that the first, listed definitional element was more strongly endorsed as a restrictive practice than the second-listed element)			
Severity of harm: *Moderate severity (cutting own body parts) OR high severity (attempting suicide)*	2 vs. 3	1.04 (0.88, 1.14), 0.949	0.91 (0.73, 1.10), 0.316	0.67 (0.52, 0.86), 0.002*	0.84 (0.64, 1.10), 0.215
Immediate complication leading to harm; *No complication (no injury) OR complication (needle stick injury to patient)*	4 vs. 5	1.14 (0.98, 1.32), 0.089	1.22 (0.89, 1.66), 0.204	0.83 (0.49, 1.40), 0.500	1.01 (0.76, 1.36), 0.896
Who implements the action; *Nurse OR security person (e.g., guard, police)*	6 vs. 7	1.11 (0.99, 1.25), 0.068	1.37 (1.06, 1.77), 0.013*	1.37 (1.04, 1.82), 0.024*	1.02 (0.75, 1.38), 0.889
Patient positioning during intervention; *Prone* OR *supine*	8 vs. 9	0.90 (0.78, 1.05), 0.221	0.91 (0.73, 1.14), 0.428	1.24 (0.73, 2.10), 0.424	0.91 (0.67, 1.24), 0.584
Door lock status; *Locked* OR *unlocked*	23 vs. 24	9.36 (6.2, 14.24), <0.001*	6.41 (3.32, 12.4), <0.001*	11.8 (4.51, 31.23), <0.001*	8.40(3.60,19.61), <0.001*
Locked door types; *Individual room doors* OR *only the whole ward door, while individuals’ room doors left open*	10 vs. 11	1.54 (1.31, 1.82), <0.001*	1.36 (1.07, 1.72), 0.001*	1.97 (1.21, 3.21), 0.006*	1.56 (1.0, 2.43), 0.047*
Consent status; *The family were not approached* OR *the family were approached, and consent was given*	14 vs. 15	0.85 (0.71, 1.01), 0.080	0.97 (0.69, 1.37), 0.895	1.91 (1.01, 3.62), 0.045*	0.79 (0.56, 1.13), 0.206
Evidence initiating the implementation of the action; *Witness attempt at physical harm OR overhear verbal threat of harm*	16 vs. 17	1.18 (0.99, 1.41), 0.064	1.05 (0.83, 1.34), 0.655	0.76 (0.50, 1.16), 0.212	0.96 (0.63, 1.45), 0.870
Evidence initiating the action; *overhear verbal threat of harm* OR *report from third party of threat of harm*	17 vs. 44	0.83 (0.63, 1.10), 0.207	1.04 (0.73, 1.48), 0.797	1.00 (0.55, 1.79), 1.00	1.43 (0.85, 2.41), 0.167
Evidence initiating the action; *Witnessing the person attempting to physically harm others* OR *report received from others stating that the person intends to physically harm others*	16 vs. 44	0.97 (0.71, 1.32), 0.868	1.08 (0.74, 1.58), 0.665	0.74 (0.38, 1.46), 0.399	1.41 (0.80, 2.49), 0.224
Duration; *Short (immediate release)* OR *long (release after one hour)*	18 vs. 19	0.67 (0.55,0.80), <0.001*	0.93 (0.68, 1.26), 0.650	0.35 (0.20, 0.60), <0.001*	0.63(0.45, 90), 0.001*
Nature of intervention; *Actual implementation* OR *only threatening with the action*	19 vs. 20	3.44 (2.74, 4.30), <0.001*	1.96 (1.37, 2.81), <0.001*	5.18 (2.75, 9.75), <0.001*	2.42 (1.60, 3.67), <0.001*
Risk assessment; *Risk assessment with high-risk harm outcome OR no risk basement conducted*	21 vs. 22	1.52 (1.23, 1.88), <0.001*	1.00 (0.72, 1.38), 0.998	0.56 (0.29, 1.10), 0.097	1.67 (1.06, 2.62), 0.025*
Trial of less restrictive alternative; *Tried, but not effective* or *not tried*	25 vs. 26	1.18 (0.89, 1.57), 0.223	1.33 (0.99, 1.79), 0.053	0.54 (0.29, 1.02), 0.060	1.29 (0.72, 2.31), 0.379
Numbers of restraint points applied on the person’s body parts; *Multiple* OR *single*	27 vs. 29	1.02 (0.82, 1.25), 0.844	1.16 (0.90,1.49), 0.236	1.03 (0.68, 1.56), 0.867	1.56 (1.11, 2.19), 0.009*
Time of the day when the action occurs; *Night time* OR *daytime*	30 vs. 31	0.94 (0.76, 1.16), 0.595	1.00 (0.76, 1.33), 0.947	0.94 (0.70, 1.25), 0.688	0.82 (0.53, 1.28), 0.411
Availability of service facilities in seclusion rooms: *Empty room OR furnished room*	32 vs. 33	1.37 (1.05, 1.80), 0.020*	1.70 (1.09, 2.64), 0.018*	1.80 (0.92, 3.48), 0.081	2.05 (1.03, 4.09), 0.041*
Legal status (medication approval): *Approved for use by hospital policy* OR *not approved*	34 vs. 35	0.92 (0.7, 1.11), 0.428	0.76 (0.58, 0.98), 0.040*	0.56 (0.32, 0.98), 0.044*	0.48 (0.28, 0.82), 0.008*
Medication dosage; *Increased dose* OR dose *in line with the hospital protocol*	34 vs. 36	1.77 (1.20, 2.61), 0.004*	1.46 (0.80, 2.66), 0.212	2.86 (1.13, 7.22), 0.026*	1.46 (0.64, 3.32), 0.362
Activities prevented during the episode of the action; Exit/*Leaving a designated area* OR *receiving visits from family, friends* or *loved ones*	37 vs. 38	1.25 (0.95, 1.65), 0.109	1.16 (0.70, 1.90), 0.555	0.84 (0.47, 1.52), 0.580	0.93 (0.53, 1.65), 0.823
Availability of other people with the person during seclusion episodes; *Alone* OR *with staff member present with the person in the room*	39 vs. 40	1.75 (1.36, 2.25), 0.001*	1.74 (1.15, 2.66), 0.009*	2.62 (1.37, 5.01), 0.003*	1.22 (0.94, 1.85), 0.107
Type of professionals presents with the person during seclusion episodes; *staff member* or *other patients*	40 vs. 41	1.11 (0.91, 1.36), 0.278	1.01 (0.75, 1.34), 0.937	0.65 (0.35, 1.20), 0.176	0.89 (0.57, 1.38), 0.603

* Indicates statistically significant differences in the classification between paired scenarios that shared identical core contexts among individuals within each participant stakeholder group.

**Table 2.** Interpretation of findings for comparisons of individuals’ classification reposes within each stakeholder group

Contextual factors/definitional elements of scenarios compared	Interpretation of findings
Severity of harm: *Moderate severity (cutting own body parts) OR high severity (attempting suicide)*	Severity of harm anticipated to be prevented was an important definitional element for service users, but not so for other stakeholders. Actions performed to prevent more severe harm were less likely to be classified as restrictive practices than actions taken to prevent less severe harm.
Immediate complication leading to harm; *No complication (no injury) OR complication (needle stick injury to patient)*	Whether there was an immediate complication or not did not impact whether a scenario was classified as a restrictive practice among any of the stakeholder groups.
Professionals implementing the action; *Nurse OR security person (e.g., guard, police)*	Having a nurse implement the action increased the likelihood that both researchers and service users classified it as a restrictive practice compared to when it was applied by a security person.
Patient positioning during intervention; *Prone* OR *supine*	Applying the action while the person was in either a prone position or supine did not impact whether the scenario was classified as a restrictive practice by any of the stakeholder groups.
Door lock status; *Locked* OR *unlocked*	Seclusion activities that involved locking a door were substantially more likely to be classified as restrictive practices compared to not locking the door across all participant groups.
Locked door types: *Individual room doors* OR *only the whole ward door, while individuals’ room doors were left open*	Locking an individual room door was more likely to be classified as a restrictive practice compared to only locking the ward door across all stakeholder groups.
Consent status; T*he family were not approached* OR *the family were approached, and consent was given*	Not approaching the family for consent was more likely to lead to the action being classified as a restrictive practice compared to approaching and receiving consent from the family, but only among service user stakeholders.
Evidence initiating the implementation of the action; *Witnessing an attempt at physical harm OR overhearing verbal threat of harm*	Whether the action was initiated after directly witnessing the person attempting to physically harm others or overhearing verbal intentions of harming others did not impact how a scenario was classified as a restrictive practice among any of the stakeholder groups.
Evidence initiating the implementation of the action; *overhearing verbal threat of harm* OR *second-hand report from a third party of threats of harming others*	Whether the action was initiated after overhearing a verbal intent of harm or based on receiving reports from others about the person’s intent to harm did not impact whether a scenario was classified as a restrictive practice among any of the stakeholder groups.
Evidence initiating the implementation of the action; *Directly witnessing the person’s attempt of harm* OR *receiving a third-party report as the person was attempting to physically harm others*	Whether the action was initiated after directly witnessing the person attempting to physically harm others or based on receiving a report from others about the person’s intent to harm did not impact whether a scenario was classified as restrictive practices among any of the stakeholder groups.
Duration; *Short (immediate release)* OR *long (release after one hour)*	Immediate release was less likely to be classified as a restrictive practice compared to those with longer durations by clinician, service user and family caregiver stakeholders, but not by researchers.
Nature of intervention; *Actual implementation* OR only *threatening the person with the implementation of the action*	Actually performing the action increased the likelihood of it being classified as a restrictive practice compared to merely threatening the person with its implementation across all participant groups.
Risk assessment; *Risk assessment with high-risk harm outcome OR no risk assessment conducted*	Performing an assessment indicating a high risk of danger before the implementation of the actions increased the likelihood of the action being classified as a restrictive practice compared to actions performed without a risk assessment, both by clinicians and family caregivers, but not among service users or researchers.
Trial of less restrictive alternative; *Tried, but not effective* or *not tried*	The trial of less restrictive alternatives that were found to be ineffective did not impact the classification of the actions as restrictive practices compared to performing the same actions without first attempting less restrictive alternatives among any of the stakeholder groups.
Numbers of restraint points applied on the person’s body parts; *Multiple* OR *single*	Having multiple restraint points on a person’s body parts compared to using a single restraint point increased the likelihood of the action being classified as restrictive practice, but only among service user stakeholders.
Time of the day when the action occurs; *Nighttime* OR *daytime*	Whether the action was performed during night-time or daytime did not have a significant impact on its classification as a restrictive practice by any of the stakeholder groups.
Availability of service facilities in seclusion rooms: *Empty room OR furnished room*	Seclusion activities performed in an empty room were more likely to be classified as restrictive practices compared to those implemented in a fully furnished room by clinicians, researchers and family caregivers, but not by service users.
Legal status (medication approval): *Approved* for use by hospital policy OR *not approved*	Administering sedative medications that are legally approved for use by the hospital was less likely to be classified as a restrictive practice compared to administering medications without hospital approval among researchers, service users, and family caregivers, but not among clinicians.
Medication dosage; *Increased dose* OR dose i*n line with the hospital protocol*	Increasing medication dosage increases the likelihood of the action being considered restrictive practices compared to dosages administered in line with hospital protocols by researcher and service user participants, but not by clinician or family caregiver stakeholders.
Activities prevented during the episode of the action; *Exit*/*Leaving a designated area* OR *receiving visits from others*	Preventing a person from leaving a designated area, compared to restricting them from receiving visits from family, friends or loved ones, did not show a statistically significant impact on classification as a restrictive practice among any of the stakeholder groups.
Availability of other people with the person during seclusion episodes; *Alone* OR *staff member present presents with the person in the seclusion room*	Seclusion episodes where individuals were left alone in a single room were more likely to be classified as restrictive practices compared to those where a staff member was present in the room among clinicians, researchers and service users, but not among family caregivers.
Persons present with the person during seclusion episodes; *staff member* or *other patients*	Whether a staff member or another patient was present with the individual during seclusion activities did not significantly impact the classification of the action as a restrictive practice by any of the stakeholder groups.

Thirteen contextual elements showed statistically significant differences in scenario classification in at least one participant group. These were: severity of anticipated harm (moderate vs. severe risk to be prevented), type of professional implementing the action (nurse vs. security staff), door lock status (door locked vs. unlocked), type of locked door (individual room door vs. only main ward door), consent status (family approached and consent given vs. family not approached), duration of the intervention episode (short vs. long), nature of intention (actual implementation of mechanical restraint vs. only threatening with mechanical restraint), risk assessment (conducted and indicating high risk vs. no risk assessment conducted), number of restraint points applied to the person’s body parts (multiple vs. a single point), seclusion room conditions (empty vs. furnished), legal status of medication (medication legally approved vs. not legally approved), medication dosage (dose in line with hospital protocol vs. increased dose beyond protocol) and presence of others during seclusion (person secluded alone vs. with a staff member present). Among these, door lock status, type of locked door, and nature of the intervention showed statistically significant variations within each of the stakeholder groups. The remaining 10 comparisons demonstrated group-specific effects, with significant differences observed only within certain stakeholder groups.

For example, the presence of another person during seclusion influenced the classification of scenarios as an RP for clinicians and researchers, but not for service users or family caregivers. Similarly, variations in duration of intervention and medication dosage showed significant differences for clinicians and service users only. Consent status and severity of harm anticipated to be prevented affected the classification patterns only among service user participants. Family caregivers were the only stakeholder group whose classification responses were significantly influenced by the number of restraint points used.

Individuals within service user groups were generally more sensitive in identifying many scenarios, including subtle forms such as verbal threats, lack of the person’s consent, and the constant presence of staff that were overlooked by other stakeholder groups. However, their scenario classification decisions shifted notably with even minor changes in scenario context descriptions (for example, locked doors versus unlocked doors, action carried out with consent versus without consent). Across all comparisons, service users’ classifications demonstrated statistically significant differences in 10 of the 22 paired scenarios comparisons, followed by clinicians and family caregivers with eight each, and researchers with seven (Tables 1 and 2).

### Scenario contexts influencing scenario classification between stakeholder groups

Contrasts in how different stakeholder groups classified the comparative case scenarios examining key contextual elements of RPs revealed that clinicians often held divergent views from service users and family caregivers and occasionally differed from researchers (Table 3).

**Table 3.** Comparisons of influence of variations in specific contextual factors/definitional elements on scenario classification across stakeholder groups: Results of interaction effect analysis

	Stakeholder group			
	Researchers	Consumers	Family caregiver	
Scenarios contrasted	Odds ratio (95% CI)			Interpretation of findings (clinicians’ classification used as the reference group)
2 vs. 3	0.89 (0.70, 1.14), 0.376	0.68 (0.51, 0.89), 0.006*	0.85 (0.65, 1.11), 0.250	Compared to clinicians, service users were less likely to classify actions applied to prevent moderate anticipated harm as restrictive practices than actions performed to prevent anticipated severe harm.
4 vs. 5	1.05 (0.75, 1.47), 0.739	0.73 (0.42, 1.59), 0.259	0.90 (0.64, 1.25), 0.540	No significant differences were observed between stakeholder groups in their classification of an action as a restrictive practice or not based on whether or not the action caused immediate complications.
6 vs. 7	1.20 (0.92, 1.57), 0.162	1.22 (0.91, 1.64), 0.181	0.90 (0.66, 1.23), 0.533	No significant differences were observed between stakeholder groups’ classification of an action as a restrictive practice or not based on whether it was applied by a ward nurse or a security person.
8 vs. 9	1.00 (0.77, 1.31), 0.954	1.37 (0.79, 2.37), 0.258	1.00 (0.72, 1.40), 0.956	No significant differences were observed between stakeholder groups in their classification of actions as restrictive practices based on whether it was applied while the person was in a prone or supine position.
23 vs. 24	0.61 (0.31, 1.19), 0.155	1.34 (0.54, 3.35), 0.522	0.75 (0.32, 1.76), 0.520	No significant differences were observed between stakeholder groups in their classification of seclusion activities as restrictive practices or not based on whether the door was locked or unlocked.
14 vs. 15	1.14 (0.76, 1.69), 0.510	2.23 (1.18, 4.21), 0.013*	0.93 (0.63, 1.39), 0.751	Compared to clinicians, service users were more likely to classify actions performed even after securing consent from family members as restrictive practices.
16 vs. 17	0.88 (0.65, 1.18), 0.406	0.64 (0.40, 1.01), 0.058	0.80 (0.53, 1.23), 0.323	No significant differences were observed between stakeholder groups in their classification of seclusion activities as restrictive practices or not based on whether individual room doors were locked or only the ward door was locked.
17 vs. 44	1.25 (0.80, 1.94), 0.313	1.20 (0.61, 1.37), 0.590	1.67 (0.95, 2.92), 0.072	No significant differences were observed between stakeholder groups in their classification of actions as restrictive practices or not based on whether the anticipated threat of harm was overheard directly or reported second-hand by a third party
16 vs. 44	1.15 (0.68, 1.77), 0.676	0.75 (0.34, 1.61), 0.464	1.43 (0.76, 2.68), 0.262	No differences were observed between stakeholder groups in their classification of actions as restrictive practices or not based on whether the threat of physical harm was directly witnessed or reported by others.
18 vs. 19	1.41 (0.98, 1.04), 0.057	0.54 (0.31, 0.95), 0.003*	1.01 (0.71, 1.44), 0.912	Compared to clinicians, service users were less likely to classify actions with short durations as restrictive practices than those with longer durations.
19 vs. 20	0.55 (0.36, 0.83), 0.005*	1.40 (0.71, 2.75), 0.323	0.59 (0.39, 0.89), 0.013*	Compared to clinicians, both researchers and service users were less likely to classify the actual implementation of restraints as restrictive practices than merely threatening the person with their use.
21 vs. 22	0.64 (0.44, 0.95), 0.028*	0.26 (0.17, 0.72), 0.004*	1.08 (0.67, 1.75), 0.736	Compared to clinicians, researchers and service users were less likely to classify actions performed after conducting a risk assessment indicating severe risk of harm as restrictive practices than actions performed without conducting a risk assessment.
25 vs. 26	1.09 (0.73,1.62), 0.650	0.44 (0.22, 0.90), 0.025*	1.12 (0.57, 2.21) 0.726	Compared to clinicians, service users were less likely to classify actions performed after the trial of less restrictive alternatives as restrictive practices than actions performed without any attempt to use less restrictive alternatives.
27 vs. 29	1.14 (0.82, 1.57), 0.421	1.01 (0.63, 1.61), 0.948	1.52 (1.03, 2.24), 0.034*	Compared to clinicians, family caregivers were more likely to classify restraint as restrictive practices when multiple restraint points were applied to a person’s body, compared to the use of a single restraint point.
30 vs. 31	1.06 (0.76, 1.49), 0.694	0.98 (0.69, 1.41), 0.941	0.88 (0.55, 1.41), 0.607	No significant differences were observed between stakeholder groups in their classification of actions as restrictive practices or not based on whether the action occurred during the daytime or at nighttime.
32 vs. 33	1.16 (0.71, 189), 0.544	1.29 (0.64, 2.61), 0.469	1.41 (0.70, 2.81), 0.321	No significant differences were observed between stakeholder groups in their classification of seclusion activities as restrictive practices or not based on whether the room was empty or furnished.
34 vs. 35	0.84 (0.62, 1.14), 0.273	0.63 (0.37, 1.07), 0.093	0.54 (0.31, 0.93), 0.028*	Compared to clinicians, family caregivers were less likely to classify the administration of hospital-approved sedative medications as restrictive practices than the use of non-approved medications.
34 vs. 36	0.77 (0.38, 1.54), 0.465	1.42 (0.61, 3.34), 0.411	0.72 (0.33, 1.55), 0.405	No differences were observed between stakeholder groups in their association on restrictive practice scenarios based on whether medication dosage was increased or administered according to hospital protocol.
37 vs. 38	0.91 (0.52, 1.59), 0.767	0.67 (0.36, 1.24), 0.206	0.75 (0.38, 1.46), 0.400	No significant differences were observed between stakeholder groups in their classification of actions as restrictive practices or not based on whether the activity involved preventing individuals from leaving a designated area or receiving visits from family, friends, or loved ones.
39 vs. 40	0.97 (0.60, 1.58), 0.926	1.46 (0.73, 2.93), 0.276	0.72 (0.49, 1.06), 1.00	No significant differences were observed between stakeholder groups in their classification of seclusion episodes as restrictive practices or not, based on whether the individual was alone in a room or accompanied by a staff member.
40 vs. 41	0.90 (0.63, 1.27), 0.555	0.59 (0.31, 1.11), 0.103	0.80 (0.53, 1.20), 0.287	No significant differences were observed between stakeholder groups in their classification of seclusion activities restrictive practices or not, based on whether a staff member or another patient was present with the individual during the episode.

* Indicates statistically significant variations in the influence of contextual factors/definitional elements on the differences observed in the classification of paired scenarios among researchers, service usersand family caregiver stakeholders compared to clinicians.

Statistically significant variations between stakeholder groups were observed in comparisons involving key contextual elements, including the severity of harm, consent status, duration of the intervention, nature of the intervention, presence of a formal risk assessment, use of less restrictive alternatives before applying RPs, number of restraint points used and legal framing of the intervention.

Service users’ responses exhibited the most frequent divergence from clinicians, with statistically significant differences identified across five contextual comparisons: severity of harm, consent status, duration of the intervention, nature of the intervention, and the use of less restrictive alternatives. Family caregivers demonstrated statistically significant differences from clinicians in three contexts (the nature of the intervention, the number of restraint points applied and the legal framing of the action). Researchers, by contrast, differed significantly from clinicians’ classifications in two contextual areas: the nature of the intervention and the presence or absence of a formal risk assessment.

## Discussion

This study revealed divergent perspectives within and between clinicians, service users, family caregivers, and researchers on how interventions are conceptualized and classified as RPs. These classification decisions were influenced more by the context in which the practice was applied, the professional role of the assessor, and their underlying epistemological orientation, rather than solely by the coercive nature of the action itself. Such inconsistencies in perspectives on RPs hinder collaborative efforts among stakeholders to implement reduction strategies effectively (Bennetts *et al.*, 2024) and undermine the consistency of rights monitoring and accountability mechanisms. This ultimately hinders the shift towards more recovery-oriented and evidence-based approaches to care (De Cuyper *et al.*, 2023), which require the active involvement of service users and their family caregivers in care planning (Slade, 2017).

A common pattern across all groups was the tendency to associate restrictiveness primarily with visible physical or environmental features like mechanical restraints or locked doors, while often overlooking more subtle actions (Series, 2015). A clear example of this is the context of door locking status: a substantial proportion of participants across all groups classified a seclusion scenario involving locked doors as an RP. However, this proportion declined markedly when the same scenario was presented without locked doors, despite the core coercive nature of the context remaining unchanged. In reality, individuals are often compelled to remain in a single room not necessarily because of physical barriers, but also due to implied negative consequences if they attempt to leave the room on their own (Klingemann *et al.*, 2022). Such forms of restriction are likely to be overlooked and left unreported, even though they can seriously limit personal freedom and carry ethical and psychological consequences comparable to more overt forms of restriction (Muluneh *et al.*, 2025).

The issue of locked doors in psychiatric settings raises critical human rights concerns due to the restriction of individual liberty, as emphasized by the Universal Declaration of Human Rights (Szmukler *et al.*, 2014). The World Health Organization and the Office of the United Nations High Commissioner for Human Rights promote open-door policies aimed at reducing coercion by balancing safety with autonomy (World Health Organization, 2023). However, the findings of this study challenge the assumption that open-door policies alone reduce coercion. Such policies may remove visible physical barriers, while subtler informal restrictions can persist if left unrecognized. This highlights that policies, classification frameworks, and clinical guidelines need to move beyond visible physical measures and give greater attention to less overt forms of restriction, ensuring that the full spectrum of RPs is accurately recognized and addressed (Beeri *et al.*, 2025).

This study highlights the stakeholder-specific nature of classification decisions, demonstrating how differences in professional roles, lived experiences, and shared assumptions shape individual perspectives on RPs (Butterworth *et al.*, 2022). Service users, in particular, showed the greatest sensitivity to contextual factors related to power, autonomy, and psychological safety, whereas most other groups relied on policies and protocols (Bennetts *et al.*, 2024). In contrast, clinicians’ classifications were more influenced by legal and procedural factors such as formal risk assessments, intervention duration, and medication dosage, often shaped by institutional protocols and medico-legal frameworks. These findings suggest that policies and protocols are likely influenced by clinician needs and preferences. This underscores the importance of valuing lived experiences and integrating them with clinical judgments to promote inpatient practices that prioritize safety, trust, and empowerment across all stakeholder groups (Forkey *et al.*, 2021).

Family caregivers generally aligned with clinicians and researchers, viewing interventions through the lens of risk mitigation rather than autonomy. This supports prior findings that family members’ views often differ from service users’ and align more with clinical staff due to safety concerns (Hupé *et al.*, 2024). These differences highlight the importance of integrating perspectives across diverse stakeholders in policy development and the implementation of reduction strategies (Molloy *et al.*, 2025).

Researchers fell between other groups, demonstrating more context sensitivity than family caregivers but less than clinicians and service users, who are often the subjects of their studies. Their classification judgments tended to rely more heavily on legal definitions and procedural guidelines, likely reflecting their familiarity with formal policy frameworks and research ethics standards, while placing less emphasis on psychological dimensions (Hupé *et al.*, 2024). Importantly, some contextual elements identified in recent systematic reviews (Hupé *et al.*, 2024; Muluneh *et al.*, 2025), as core conceptual themes for defining RPs, such as the time of day, the activity the person was engaged in during the episode, and whether less restrictive alternatives were attempted did not influence how RPs were actually interpreted by any of the stakeholder groups. The disconnect between academic theory and real-world decision-making highlights the limitations of relying solely on theory-derived definitions and classification frameworks (Belayneh *et al.*, 2019), which may not fully capture how RPs are perceived and experienced in clinical practice (Hupé *et al.*, 2024). This underscores the need to empirically validate such systems in real-world settings to ensure their effectiveness and applicability.

### Implications

Diverging views on what constitutes an RP among stakeholders complicate collaborative decision-making and hinder the consistent application of least-restrictive alternatives (Rose *et al.*, 2017). These discrepancies affect consistency in the implementation, monitoring and evaluation (Janssen *et al.*, 2011) of reduction strategies in mental health care and the integration of least restrictive alternatives in clinical practice (Muluneh *et al.*, 2025). Practices that one healthcare professional considers least restrictive may be perceived as restrictive or even harmful by others (Bennetts *et al.*, 2024) or experienced as intrusive by service users. Therefore, clinicians are required to thoughtfully balance or integrate different viewpoints with their clinical judgment to reach a workable approach when implementing RP reduction strategies and selecting the least restrictive alternatives (Duffy *et al.*, 2023). Such inclusive approaches ensure practices are conceptually coherent, ethically grounded, and practically applicable across diverse settings (Rose *et al.*, 2017).

Inconsistencies in how RPs are defined also affect the validity and comparability of research. For example, researchers analyzing hospital records may use criteria that differ from those applied in clinical incident reporting systems. Similarly, when data are collected by asking service users if they can recall whether and how frequently they experienced RPs during their hospital stay, their responses may be distorted by differences in how they interpret what constitutes RPs. Such variabilities can introduce systematic bias, ultimately limiting the utility of research findings for benchmarking, evaluating trends, and guiding quality improvement efforts (Haines *et al.*, 2008). Researchers are encouraged to triangulate data from multiple sources, such as medical records, incident reports, and routine surveys of staff and service users. This can help lead to a more comprehensive and reliable evidence base, reducing underreporting and capturing informal or undocumented practices that might otherwise be overlooked by using individual methods (Hallett and McLaughlin, 2022).

The overall findings underscore the need for harmonized definitions and classification systems for RPs. Achieving consensus across stakeholders is a long-term process and may be challenging. Even providing a written definition for healthcare practices does not necessarily mean stakeholder agreement on the definition and classification will improve (Haines *et al.*, 2009). Variations in perspectives on RPs between stakeholders and across regions, informed by cultural differences, educational backgrounds, and local policy frameworks, are likely to pose challenges to the harmonization process. These efforts may be more effective if supported by intermediate and complementary strategies such as the development of robust monitoring systems and educational programs (Janssen *et al.*, 2011). Ongoing capacity-building initiatives, such as therapeutic patient education and rights-based programs like the WHO’s Quality Rights initiative (World Health Organization, 2019) can further support progress towards harmonization (Haines *et al.*, 2009; Lickiewicz *et al.*, 2024).

### Limitations

The use of hypothetical scenarios may not adequately capture individuals’ interpretations of whether an action constitutes an RP in clinical settings. In reality, such judgments are often shaped by contextual pressures, emotional responses, institutional dynamics, and organizational stress. These factors may not be fully simulated through scenario vignettes. However, the use of standardized cases facilitated meaningful comparisons across participants and regions while also strengthening internal validity. Moreover, the engagement of diverse mental health stakeholders in the co-design of these scenarios enriched the study by incorporating a wide range of perspectives and contexts.

Participants in this study were drawn from a variety of professional backgrounds across 46 countries worldwide. However, some countries and regions contributed larger numbers of responses than others, with a small number of responses from North and South America. This may bias our overall results towards the responses of regions with more respondents. The legal frameworks and cultural factors may influence the classification and reporting of RPs in these regions in particular and may lead to different results compared to what we have observed in this study. Additionally, a relatively small sample of service users (*n* = 80) compared to clinicians (*n* = 517) may have reduced statistical power in some subgroup comparisons, potentially limiting the robustness of inferences drawn from service user perspectives. The purposive and snowball sampling techniques may also have introduced selection bias, as participants were likely those with a particular interest or experience in RPs, which could affect the representativeness of the findings.

The varying perspectives of mental health stakeholder groups towards RPs may influence their interpretation of case scenarios. These perspectives can be shaped by differences in training levels, cultural norms across regions, local policies and laws, disciplinary backgrounds, and prior exposure to RPs and mental health care. This highlights the need for future research to explore whether and how these factors influence stakeholders’ views regarding whether or not a certain action constitutes RP. Employing mixed-methods designs or subgroup analyses could help better understand these influences, thereby strengthening the evidence base to endorse and apply for internationally acceptable, stakeholder-informed RP frameworks.

## Conclusions

This study underscores the need for internationally harmonized definitions and standardized classification systems for RPs, co-designed with diverse stakeholders, including clinicians, service users, family caregivers and researchers. Without such frameworks, efforts to measure, monitor and reduce RPs risk being undermined by inconsistent understanding and documentation. Embedding these definitions into training, reporting systems and research tools will support valid comparisons across settings, foster ethical and rights-based care, and ensure that reform efforts proceed with clarity, accountability and meaningful impact.

## Supporting information

10.1017/S2045796025100322.sm001 Belayneh et al. supplementary material 1 Belayneh et al. supplementary material

10.1017/S2045796025100322.sm002 Belayneh et al. supplementary material 2 Belayneh et al. supplementary material

## Data Availability

The de-identified raw data for this manuscript are available and can be accessed from the corresponding author upon request at zelalem.muluneh@monash.edu or zelalembe45@gmail.com.

## Supplementary material

The supplementary material for this article can be found at https://doi.org/10.1017/S2045796025100322.

## References

[ref1] Beeri S, Baumberger E, Zwakhalen S and Hahn S (2025). Conceptualisation of informal coercion in inpatient psychiatry: a scoping review. *International Journal of Mental Health Nursing* 34(3), e70076. 40474445 10.1111/inm.70076

[ref2] Belayneh Z, Abebaw D, Amare T, Haile K and Abebe Z (2019). Perception regarding the causes of schizophrenia and associated factors among feresbet district residents: a community based study. *BMC Public Health* 19, 1–7. 30909977 10.1186/s12889-019-6678-4 PMC6434636

[ref3] Belayneh Z, Chavulak J, Lee DCA, Petrakis M and Haines TP (2024). Prevalence and variability of restrictive care practice use (physical restraint, seclusion and chemical restraint) in adult mental health inpatient settings: a systematic review and meta‐analysis. *Journal of Clinical Nursing* 33(4), 1256–1281. 38304928 10.1111/jocn.17041

[ref4] Belayneh Z, Chavulak J, Lee DCA, Petrakis M and Haines TP (2025a). Methodological issues in measuring restrictive care practices (Mechanical/physical restraint, chemical restraint and seclusion) in adult mental health inpatient units: a systematic review of recent literature. *Journal of Clinical Nursing* 34(5), 1629–1647. 39653688 10.1111/jocn.17588

[ref5] Belayneh Z, Lee DCA, Haines TP, Aluh DO, Onu JU, Newton‐Howes G, Masters K, Kohn Y, Sin J and Goulet MH (2025b). Co‐designing case scenarios and survey strategies to examine the classification and reporting of restrictive care practices in adult mental health inpatient settings: perspectives from international stakeholders. *International Journal of Mental Health Nursing* 34(1), e13479. 39629922 10.1111/inm.13479

[ref6] Bennetts SL, Pepin G, Moylan S, Carolin R and Lucas J (2024). Elimination of restrictive practices from acute adult mental health care services: a qualitative evidence synthesis of the lived experience literature. *SSM-Mental Health* 5, 100305.

[ref7] Boas TC, Christenson DP and Glick DM (2020). Recruiting large online samples in the United States and India: facebook, mechanical Turk, and qualtrics. *Political Science Research and Methods* 8(2), 232–250.

[ref9] Butterworth H, Wood L and Rowe S (2022). Patients’ and staff members’ experiences of restrictive practices in acute mental health in-patient settings: systematic review and thematic synthesis. *BJPsych Open* 8(6), e178. 36200350 10.1192/bjo.2022.574 PMC9634587

[ref10] Carter B and Ponte AD (2022). Integrating web applications into popular survey platforms for online experiments. *Behavior Research Methods* 54(6), 3093–3099. 35212937 10.3758/s13428-022-01792-w

[ref11] Chieze M, Hurst S, Kaiser S and Sentissi O (2019). Effects of seclusion and restraint in adult psychiatry: a systematic review. *Frontiers in Psychiatry* 10, 491. 31404294 10.3389/fpsyt.2019.00491 PMC6673758

[ref12] De Cuyper K, Vanlinthout E, Vanhoof J, van Achterberg T, Opgenhaffen T, Nijs S, Peeters T, Put J, Maes B and Van Audenhove C (2023). Best practice recommendations on the application of seclusion and restraint in mental health services: an evidence, human rights and consensus‐based approach. *Journal of Psychiatric and Mental Health Nursing* 30(3), 580–593. 36565433 10.1111/jpm.12890

[ref13] Duffy J, Boyle S and Del Villar K (2023). What does ‘Least Restrictive’ or ‘Less Restrictive’ mean in mental health law? Contradictions and confusion in the case of Queensland, Australia. *American Journal of Law & Medicine* 49(2-3), 286–300. 38344792 10.1017/amj.2023.32

[ref14] Forkey H, Szilagyi M, Kelly ET and Duffee J (2021). Trauma-informed care. *Pediatrics* 148(2), 15–21. 10.1542/peds.2021-052579 34312294

[ref15] Grace-Martin K (2020). Strategies for choosing the reference category in dummy coding. Retrieved January 20.

[ref16] Haines TP, Cornwell P, Fleming J, Varghese P and Gray L (2008). Documentation of in-hospital falls on incident reports: qualitative investigation of an imperfect process. *BMC Health Services Research* 8(1), 254. 19077252 10.1186/1472-6963-8-254 PMC2621198

[ref17] Haines TP, Massey B, Varghese P, Fleming J and Gray L (2009). Inconsistency in classification and reporting of in‐hospital falls. *Journal of the American Geriatrics Society* 57(3), 517–523. 19187413 10.1111/j.1532-5415.2008.02142.x

[ref18] Hallett N and McLaughlin P (2022). Restrictive interventions: understanding and reducing their use in mental health settings. *Mental Health Practice* 25(6), 34–41.

[ref20] Hupé C, Larue C and Contandriopoulos D (2024). Defining chemical restraint: a preliminary step towards measurement and quality assessment. *Aggression and Violent Behavior* 77, 101926.

[ref21] Janssen W, van de Sande R, Noorthoorn E, Nijman H, Bowers L, Mulder C, Smit A, Widdershoven GA and Steinert T (2011). Methodological issues in monitoring the use of coercive measures. *International Journal of Law and Psychiatry* 34(6), 429–438. 22079087 10.1016/j.ijlp.2011.10.008

[ref22] Jungfer H-A, Schneeberger AR, Borgwardt S, Walter M, Vogel M, Gairing SK, Lang UE and Huber CG (2014). Reduction of seclusion on a hospital-wide level: successful implementation of a less restrictive policy. *Journal of Psychiatric Research* 54, 94–99. 24726637 10.1016/j.jpsychires.2014.03.020

[ref23] Klingemann J, Świtaj P, Lasalvia A and Priebe S (2022). Behind the screen of voluntary psychiatric hospital admissions: a qualitative exploration of treatment pressures and informal coercion in experiences of patients in Italy, Poland and the United Kingdom. *International Journal of Social Psychiatry* 68(2), 457–464. 33855874 10.1177/00207640211003942

[ref24] Lickiewicz J, Efkemann SA, Husum TL, Lantta T, Pingani L and Whittington R (2024). Expert opinions on improving coercion data collection across Europe: a concept mapping study. *Frontiers in Psychiatry* 15, 1403094. 38868490 10.3389/fpsyt.2024.1403094 PMC11167108

[ref25] Maguire T, Ryan J, Fullam R and McKenna B (2018). Evaluating the introduction of the safewards model to a medium-to long-term forensic mental health ward. *Journal of Forensic Nursing* 14(4), 214–222. 30433910 10.1097/JFN.0000000000000215

[ref26] Mayers P, Keet N, Winkler G and Flisher AJ (2010). Mental health service users’ perceptions and experiences of sedation, seclusion and restraint. *International Journal of Social Psychiatry* 56(1), 60–73. 20053723 10.1177/0020764008098293

[ref27] McSherry B (2013). The legal regulation of seclusion and restraint in mental health facilities. *Journal of Law and Medicine* 21(2), 251–254. 24597370

[ref28] Molloy N, Kilcoyne I, Belcher H and Wykes T (2025). Exploring the involvement of people with lived experience of mental disorders in co-developing outcome measures: a systematic review. *The Lancet Psychiatry* 12(2), 140–152. 39848731 10.1016/S2215-0366(24)00376-6

[ref29] Molnar A (2019). SMARTRIQS: a simple method allowing real-time respondent interaction in Qualtrics surveys. *Journal of Behavioral and Experimental Finance* 22, 161–169.

[ref30] Muluneh ZB, Chavulak J, Lee D-CA, Petrakis M and Haines TP (2025). Variations in definitions used for describing restrictive care practices (seclusion and restraint) in adult mental health inpatient units: a systematic review and content analysis. *Social Psychiatry & Psychiatric Epidemiology* 60(1), 1–24. 39080007 10.1007/s00127-024-02739-6 PMC11790767

[ref31] Murphy J, Qureshi O, Endale T, Esponda GM, Pathare S, Eaton J, De Silva M and Ryan G (2021). Barriers and drivers to stakeholder engagement in global mental health projects. *International Journal of Mental Health Systems* 15(1), 30. 33812375 10.1186/s13033-021-00458-y PMC8019163

[ref8] Paterson B, Bennet L and Bradley P (2014). Positive and Proactive Care: could new guidance lead to more problems? *British Journal of Nursing* 23(17), 939–941. 25251176 10.12968/bjon.2014.23.17.939

[ref32] Power T, Baker A and Jackson D (2020). Only ever as a last resort’: mental health nurses’ experiences of restrictive practices. *International Journal of Mental Health Nursing* 29(4), 674–684. 32048469 10.1111/inm.12701

[ref33] Riahi S, Dawe IC, Stuckey MI and Klassen PE (2016). Implementation of the six core strategies for restraint minimization in a specialized mental health organization. *Journal of Psychosocial Nursing and Mental Health Services* 54(10), 32–39. 27699424 10.3928/02793695-20160920-06

[ref34] Rose D, Perry E, Rae S and Good N (2017). Service user perspectives on coercion and restraint in mental health. *BJPsych International* 14(3), 59–61. 10.1192/s2056474000001914 29093946 PMC5618900

[ref35] Series L (2015). Relationships, autonomy and legal capacity: mental capacity and support paradigms. *International Journal of Law and Psychiatry* 40, 80–91. 25982964 10.1016/j.ijlp.2015.04.010

[ref36] Slade M (2017). Implementing shared decision making in routine mental health care. *World Psychiatry: Official Journal of the World Psychiatric Association (WPA)* 16(2), 146–153. 28498575 10.1002/wps.20412 PMC5428178

[ref38] Szmukler G, Daw R and Callard F (2014). Mental health law and the UN Convention on the rights of persons with disabilities. *International Journal of Law and Psychiatry* 37(3), 245–252. 24280316 10.1016/j.ijlp.2013.11.024 PMC4024199

[ref39] World Health Organization. (2019). *Strategies to end seclusion and restraint: WHO QualityRights Specialized training: Course slides* (WHO/MSD/QR/19.7). World Health Organization. https://www.who.int/publications/i/item/9789241516754 (accessed 30 June 2019).

[ref40] World Health Organization, and Office of the United Nations High Commissioner for Human Rights (2023). *Mental Health, Human Rights and Legislation: Guidance and Practice*. World Health Organization. https://www.who.int/publications/i/item/9789240080737 (accessed 9 October 2023).

