# Supplementary material for: Diverging views between clinicians, service users, family caregivers and researchers on the classification of restrictive practices in mental health services
Source: Epidemiol Psychiatr Sci. 2025 Dec 12;34:e59. doi: 10.1017/S2045796025100322 (PMC12721989; doi:10.1017/S2045796025100322)
Supplement: Belayneh et al. supplementary material 1 — Belayneh et al. supplementary material [file S2045796025100322sup001.docx]

**Supplementary File 2:** Frequency and proportion of participant responses for the classification of each of the 44 case scenarios whether or not as restricitve practice, cross-tabulated across stakeholder group

|  | **Outcome question**: Do you think that this scenario describes a restrictive care practice? | | | | |
| --- | --- | --- | --- | --- | --- |
| Participant group | **Response options** | | | | |
|  | Definitely yes n (%) | Probably yes n (%) | Probably no n (%) | Definitely no n (%) | I do not want to respond to this question n (%) |
| **Scenario #1:** A nurse forcefully confines a person in a locked room to minimize the risk of harm to others | | | | | |
| Clinicians | 331 (63.29) | 113 (21.61) | 37 (7.07) | 39 (7.46) | 3 (0.57) |
| Researchers | 86 (55.13) | 38 (24.36) | 13 (8.33) | 18 (11.54) | 1 (0.64) |
| Service users | 49 (70.00) | 14 (20.00) | 2 (2.86) | 4 (5.71) | 1 (1.43) |
| Family caregivers | 39 (41.05) | 32 (33.68) | 13 (13.68) | 9 (9.47) | 2 (2.11) |
| **Scenario #2:** A nurse forcefully confines a person in a locked room to minimize the risk of self-harm through cutting of body parts | | | | | |
| Clinicians | 312 60.94) | 89 (17.38) | 45 (8.79) | 63 (12.30) | 3 (0.59) |
| Researchers | 74 (48.37) | 37 (24.18) | 14 (9.15) | 27 (17.65) | 1 (0.65) |
| Service users | 41 (60.29) | 15 (22.06) | 4 (5.88) | 8 (11.76) | 0 (0.00) |
| Family caregivers | 42 (45.16) | 26 (27.96) | 13 (13.98) | 10 (10.75) | 2 (2.15) |
| **Scenario #3:** A nurse forcefully confines a person in a locked room to minimize the risk of suicide | | | | | |
| Clinicians | 304(60.80) | 89 (17.80) | 43 (8.60) | 61 (12.20) | 3 (0.60) |
| Researchers | 77 (50.33) | 37 (24.18) | 16 (10.46) | 22 (14.38) | 1 (0.65) |
| Service users | 46 (67.65) | 16 (23.53) | 2 (2.94) | 4 (5.88) | 0 (0.00) |
| Family caregivers | 45 (50.00) | 24 (26.67) | 13 (14.44) | 8 (8.89) | 0 (0.00) |
| **Scenario #4:** A person refuses to take prescribed medication so two nurses hold the person onto a bed to facilitate administration of the medication. The medication is safely administered | | | | | |
| Clinicians | 288(58.42) | 103(20.89) | 55 (11.16) | 45 (9.13) | 2 (0.41) |
| Researchers | 77 (50.66) | 39 (25.66) | 22 (14.47) | 13 (8.55) | 1 (0.66) |
| Service users | 51 (75.00) | 9 (13.24) | 4 (5.88) | 3 (4.41) | 1 (1.47) |
| Family caregivers | 41 (45.56) | 27 (30.00) | 11 (12.22) | 10 (11.11) | 1 (1.11) |
| **Scenario #5:** A person refuses to take prescribed medication so two nurses hold the person onto a bed to facilitate administration of the medication. The medication is administered but the patient sustains a needle injury during the process, | | | | | |
| Clinicians | 273(55.71) | 97 (19.80) | 64 (13.06) | 53 (10.82) | 3 (0.61) |
| Researchers | 73 (48.67) | 29 (19.33) | 29 (19.33) | 18 (12.00) | 1 (0.67) |
| Service users | 53 (80.30) | 5 (7.58) | 5 (7.58) | 3 (4.55) | 0 (0.00) |
| Family caregivers | 45 (50.00) | 16 (17.78) | 11 (12.22) | 16 (17.78) | 2 (2.22) |
| **Scenario #6:** The person is displaying fearful behaviour by punching the walls of the room. To minimize the risk of self-harm, a security person (guard) firmly holds the person's arm | | | | | |
| Clinicians | 237(48.87) | 128(26.39) | 69 (14.23) | 49 (10.10) | 2 (0.41) |
| Researchers | 59 (39.60) | 52 (34.90) | 24 (16.11) | 13 (8.72) | 1 (0.67) |
| Service users | 38 (57.58) | 17 (25.76) | 8 (12.12) | 2 (3.03) | 1 (1.52) |
| Family caregivers | 29 (32.22) | 30 (33.33) | 17 (18.89) | 14 (15.56) | 0 (0.00) |
| **Scenario #7:** The person is displaying fearful behaviour by punching the wall of his room. To minimize the risk of self-harm, a nurse firmly holds the person's arm | | | | | |
| Clinicians | 214(45.34) | 132(27.97) | 83 (17.58) | 41 (8.69) | 2 (0.42) |
| Researchers | 52 (34.90) | 43 (28.86) | 37 (24.83) | 16 (10.74) | 1 (0.67) |
| Service users | 33 (51.56) | 15 (23.44) | 12 (18.75) | 3 (4.69) | 1 (1.56) |
| Family caregivers | 26 (28.89) | 33 (36.67) | 21 (23.33) | 10 (11.11) | 0 (0.00) |
| **Scenario #8:** A nurse applies mechanical restraints to a person by keeping the person lying flat on his/her stomach, with his/her face and chest pointing downwards (prone position) | | | | | |
| Clinicians | 304(65.80) | 71 (15.37) | 36 (7.79) | 46 (9.96) | 5 (1.08) |
| Researchers | 87 (58.39) | 17 (11.41) | 25 (16.78) | 17 (11.41) | 3 (2.01) |
| Service users | 54 (85.71) | 3 (4.76) | 3 (4.76) | 2 (3.17) | 1 (1.59) |
| Family caregivers | 47 (52.22) | 16 (17.78) | 11 (12.22) | 14 (15.56) | 2 (2.22) |
| **Scenario #9:** A nurse applies mechanical restraints to a person by keeping the person lying flat on his/her back, with his/her face and chest pointing upwards (supine position) | | | | | |
| Clinicians | 303(65.87) | 96 (20.87) | 38 (8.26) | 20 (4.35) | 3 (0.65) |
| Researchers | 83 (56.08) | 40 (27.03) | 12 (8.11) | 12 (8.11) | 1 (0.68) |
| Service users | 52 (82.54) | 6 (9.52) | 3 (4.76) | 1 (1.59) | 1 (1.59) |
| Family caregivers | 43 (47.78) | 28 (31.11) | 7 (7.78) | 7 (7.78) | 5 (5.56) |
| **Scenario #10:** A nurse locks the individual person's room door to prevent him/her from escaping the hospital | | | | | |
| Clinicians | 275(60.31) | 93 (20.39) | 47 (10.31) | 38 (8.33) | 3 (0.66) |
| Researchers | 71 (47.97) | 38 (25.68) | 23 (15.54) | 15 (10.14) | 1 (0.68) |
| Service users | 43 (69.35) | 11 (17.74) | 6 (9.68) | 1 (1.61) | 1 (1.61) |
| Family caregivers | 45 (50.56) | 27 (30.34) | 8 (8.99) | 8 (8.99) | 1 (1.12) |
| **Scenario #11:** A nurse locks the whole ward door to prevent a person from escaping the hospital. | | | | | |
| Clinicians | 227(50.00) | 99 (21.81) | 65 (14.32) | 59 (13.00) | 4 (0.88) |
| Researchers | 64 (43.24) | 30 (20.27) | 31 (20.95) | 21 (14.19) | 2 (1.35) |
| Service users | 36 (58.06) | 6 (9.68) | 15 (24.19) | 4 (6.45) | 1 (1.61) |
| Family caregivers | 36 (40.45) | 26 (29.21) | 15 (16.85) | 10 (11.24) | 2 (2.25) |
| **Scenario #12:** A person is kept under mechanical restraint while being transferred between wards to promote safety. | | | | | |
| Clinicians | 284 (62.97) | 95 (21.06) | 37 (8.20) | 32 (7.10) | 3 (0.67) |
| Researchers | 70 (47.30) | 41 (27.70) | 23 (15.54) | 12 (8.11) | 2 (1.35) |
| Service users | 50 (80.65) | 5 (8.06) | 5 (8.06) | 1 (1.61) | 1 (1.61) |
| Family caregivers | 37 (42.05) | 33 (37.50) | 9 (10.23) | 6 (6.82) | 3 (3.41) |
| **Scenario #13:** A person is kept under mechanical restraint when staff feel overloaded during busy days to promote safety. | | | | | |
| Clinicians | 281(62.72) | 57 (12.72) | 39 (8.71) | 64 (14.29) | 7 (1.56) |
| Researchers | 84 (57.14) | 17 (11.56) | 20 (13.61) | 25 (17.01) | 1 (0.68) |
| Service users | 52 (83.87) | 3 (4.84) | 4 (6.45) | 3 (4.84) | 0 (0.00) |
| Family caregivers | 44 (50.57) | 12 (13.79) | 10 (11.49) | 20 (22.99) | 1 (1.15) |
| **Scenario #14:** Nurses discuss use of mechanical restraints with the person, but the person refused to give consent to this action. Then, these nurses apply mechanical restraints to the person, | | | | | |
| Clinicians | 293(66.44) | 77 (17.46) | 38 (8.62) | 31 (7.03) | 2 (0.45) |
| Researchers | 89 (60.96) | 20 (13.70) | 18 (12.33) | 16 (10.96) | 3 (2.05) |
| Service users | 52 (83.87) | 5 (8.06) | 4 (6.45) | 0 (0.00) | 1 (1.61) |
| Family caregivers | 40 (45.98) | 14 (16.09) | 16 (18.39) | 15 (17.24) | 2 (2.30) |
| **Scenario #15:** Nurses discuss use of mechanical restraints with the person, but the person refused to give consent to this action. These nurses later engaged in a discussion with the person's family member, and the family member granted consent. The nurses then apply mechanical restraints to the person | | | | | |
| Clinicians | 298(68.51) | 85 (19.54) | 30 (6.90) | 18 (4.14) | 4 (0.92) |
| Researchers | 83 (56.85) | 39 (26.71) | 12 (8.22) | 10 (6.85) | 2 (1.37) |
| Service users | 44 (73.33) | 10 (16.67) | 4 (6.67) | 1 (1.67) | 1 (1.67) |
| Family caregivers | 39 (44.83) | 26 (29.89) | 13 (14.94) | 7 (8.05) | 2 (2.30) |
| **Scenario #16:** A nurse forcefully confines a person to a seclusion room after witnessing the person attempting to physically harm others. | | | | | |
| Clinicians | 293 (67.98) | 76 (17.63) | 40 (9.28) | 21 (4.87) | 1 (0.23) |
| Researchers | 77 (52.74) | 44 (30.14) | 13 (8.90) | 10 (6.85) | 2 (1.37) |
| Service users | 39 (65.00) | 9 (15.00) | 8 (13.33) | 2 (3.33) | 2 (3.33) |
| Family caregivers | 37 (43.02) | 27 (31.40) | 12 (13.95) | 8 (9.30) | 2 (2.33) |
| **Scenario #17:** A nurse forcefully confines a person to a seclusion room after overhearing the person verbally expressing an intention to physically harm others | | | | | |
| Clinicians | 277(64.42) | 73 (16.98) | 49 (11.40) | 26 (6.05) | 5 (1.16) |
| Researchers | 78 (53.79) | 34 (23.45) | 20 (13.79) | 10 (6.90) | 3 (2.07) |
| Service users | 42 (70.00) | 9 (15.00) | 5 (8.33) | 3 (5.00) | 1 (1.67) |
| Family caregivers | 40 (46.51) | 23 (26.74) | 18 (20.93) | 5 (5.81) | 0 (0.00) |
| **Scenario #18:** The medical team applies mechanical restraints to safely administer injectable medications to a person who exhibits fearful behaviours during hospital admission. The team releases the mechanical restraints immediately after administering the injection. | | | | | |
| Clinicians | 258(60.42) | 81 (18.97) | 43 (10.07) | 38 (8.90) | 7 (1.64) |
| Researchers | 71 (48.97) | 33 (22.76) | 27 (18.62) | 12 (8.28) | 2 (1.38) |
| Service users | 38 (63.33) | 11 (18.33) | 9 (15.00) | 1 (1.67) | 1 (1.67) |
| Family caregivers | 28 (32.94) | 35 (41.18) | 11 (12.94) | 8 (9.41) | 3 (3.53) |
| **Scenario #19:** The medical team applies mechanical restraints to safely administer injectable medications to a person who exhibits fearful behaviours during hospital admission. The team decided to keep the person restrained for one hour after administering the injection. | | | | | |
| Clinicians | 294(69.50) | 75 (17.73) | 29 (6.86) | 22 (5.20) | 3 (0.71) |
| Researchers | 73 (50.34) | 39 (26.90) | 17 (11.72) | 15 (10.34) | 1 (0.69) |
| Service users | 49 (81.67) | 8 (13.33) | 3 (5.00) | 0 (0.00) | 0 (0.00) |
| Family caregivers | 40 (47.06) | 24 (28.24) | 14 (16.47) | 4 (4.71) | 3 (3.53) |
| **Scenario #20:** The medical team threatens the use of mechanical restraint to safely administer injectable medications to a person who exhibits fearful behaviour during hospital admission. | | | | | |
| Clinicians | 168(40.10) | 101 (24.11) | 85 (20.29) | 58 (13.84) | 7 (1.67) |
| Researchers | 49 (34.27) | 39 (27.27) | 32 (22.38) | 22 (15.38) | 1 (0.70) |
| Service users | 29 (48.33) | 13 (21.67) | 15 (25.00) | 3 (5.00) | 0 (0.00) |
| Family caregivers | 21 (24.71) | 31 (36.47) | 23 (27.06) | 8 (9.41) | 2 (2.35) |
| **Scenario #21:** A nurse uses belts to secure a person's arms and legs to the bed as a safety measure to prevent self-harm. This action is taken based on the risk assessment that indicates a higher risk of danger for this person | | | | | |
| Clinicians | 286(68.92) | 64 (15.42) | 40 (9.64) | 20 (4.82) | 5 (1.20) |
| Researchers | 81 (56.64) | 28 (19.58) | 20 (13.99) | 12 (8.39) | 2 (1.40) |
| Service users | 45 (76.27) | 4 (6.78) | 7 (11.86) | 1 (1.69) | 2 (3.39) |
| Family caregivers | 43 (50.59) | 26 (30.59) | 11 (12.94) | 3 (3.53) | 2 (2.35) |
| **Scenario #22:** A nurse uses belts to secure a person's arms and legs to the bed as a safety measure to prevent self-harm. This action is taken without conducting a risk assessment for this person | | | | | |
| Clinicians | 260(62.80) | 44 (10.63) | 45 (10.87) | 61 (14.73) | 4 (0.97) |
| Researchers | 87 (60.84) | 13 (9.09) | 22 (15.38) | 19 (13.29) | 2 (1.40) |
| Service users | 51 (86.44) | 2 (3.39) | 3 (5.08) | 3 (5.08) | 0 (0.00) |
| Family caregivers | 41 (48.24) | 11 (12.94) | 9 (10.59) | 22 (25.88) | 2 (2.35) |
| **Optional scenarios** | | | | | |
| **Scenario #23:** A person is led to a single room to prevent self-harm, and the door is locked. | | | | | |
| Clinicians | 144(66.06) | 37 (16.97) | 15 (6.88) | 22 (10.09) | 0 (0.00) |
| Researchers | 52 (55.32) | 16 (17.02) | 13 (13.83) | 12 (12.77) | 1 (1.06) |
| Service users | 30 (68.18) | 8 (18.18) | 2 (4.55) | 2 (4.55) | 2 (4.55) |
| Family caregivers | 29 (58.00) | 9 (18.00) | 9 (18.00) | 3 (6.00) | 0 (0.00) |
| **Scenario #24:** A person is led to a single room to prevent self-harm, but the door is left unlocked | | | | | |
| Clinicians | 30 (13.82) | 39 (17.97) | 85 (39.17) | 63 (29.03) | 0 (0.00) |
| Researchers | 8 (8.51) | 20 (21.28) | 41 (43.62) | 24 (25.53) | 1 (1.06) |
| Service users | 8 (18.60) | 7 (16.28) | 12 (27.91) | 15 (34.88) | 1 (2.33) |
| Family caregivers | 4 (8.00) | 14 (28.00) | 16 (32.00) | 16 (32.00) | 0 (0.00) |
| **Scenario #25:** The medical team uses devices to restrain a person who is displaying fearful behaviour. The team initially tried sedative medications, but they were not effective. | | | | | |
| Clinicians | 145(67.44) | 45 (20.93) | 17 (7.91) | 8 (3.72) | 0 (0.00) |
| Researchers | 52 (55.32) | 29 (30.85) | 9 (9.57) | 2 (2.13) | 2 (2.13) |
| Service users | 30 (69.77) | 6 (13.95) | 4 (9.30) | 2 (4.65) | 1 (2.33) |
| Family caregivers | 29 (58.00) | 14 (28.00) | 3 (6.00) | 4 (8.00) | 0 (0.00) |
| **Scenario #26:** The medical team uses devices to restrain a person who is displaying fearful behaviour. The team thinks that this is the only option to achieve the desired outcome for that case, but they did not try other approaches first. | | | | | |
| Clinicians | 143(67.45) | 20 (9.43) | 29 (13.68) | 20 (9.43) | 0 (0.00) |
| Researchers | 52 (55.32) | 14 (14.89) | 16 (17.02) | 11 (11.70) | 1 (1.06) |
| Service users | 35 (81.40) | 2 (4.65) | 5 (11.63) | 1 (2.33) | 0 (0.00) |
| Family caregivers | 29 (58.00) | 6 (12.00) | 6 (12.00) | 9 (18.00) | 0 (0.00) |
| **Scenario #27:** A nurse applies chain restraints by securing both the person’s wrists and ankles to a bed, | | | | | |
| Clinicians | 152(72.38) | 27 (12.86) | 12 (5.71) | 18 (8.57) | 1 (0.48) |
| Researchers | 61 (64.89) | 13 (13.83) | 9 (9.57) | 9 (9.57) | 2 (2.13) |
| Service users | 34 (79.07) | 5 (11.63) | 2 (4.65) | 2 (4.65) | 0 (0.00) |
| Family caregivers | 33 (66.00) | 9 (18.00) | 5 (10.00) | 3 (6.00) | 0 (0.00) |
| **Scenario #28:** A nurse applies chain restraints by securing one of the person's wrists and ankles to a bed. | | | | | |
| Clinicians | 151(71.90) | 33 (15.71) | 11 (5.24) | 13 (6.19) | 2 (0.95) |
| Researchers | 56 (59.57) | 18 (19.15) | 9 (9.57) | 8 (8.51) | 3 (3.19) |
| Service users | 34 (79.07) | 4 (9.30) | 2 (4.65) | 3 (6.98) | 0 (0.00) |
| Family caregivers | 28 (56.00) | 9 (18.00) | 5 (10.00) | 6 (12.00) | 2 (4.00) |
| **Scenario #29:** A nurse applies chain restraints by securing one of the person’s wrists to a bed. | | | | | |
| Clinicians | 148(70.81) | 34 (16.27) | 8 (3.83) | 17 (8.13) | 2 (0.96) |
| Researchers | 57 (60.64) | 21 (22.34) | 6 (6.38) | 8 (8.51) | 2 (2.13) |
| Service users | 34 (79.07) | 4 (9.30) | 2 (4.65) | 3 (6.98) | 0 (0.00) |
| Family caregivers | 28 (56.00) | 10 (20.00) | 5 (10.00) | 6 (12.00) | 1 (2.00) |
| **Scenario #30:** A nurse securely locks the door of the person's room as a safety measure during the nighttime | | | | | |
| Clinicians | 131(62.98) | 38 (18.27) | 23 (11.06) | 16 (7.69) | 0 (0.00) |
| Researchers | 45 (47.87) | 24 (25.53) | 19 (20.21) | 5 (5.32) | 1 (1.06) |
| Service users | 29 (67.44) | 6 (13.95) | 4 (9.30) | 3 (6.98) | 1 (2.33) |
| Family caregivers | 23 (46.00) | 15 (30.00) | 10 (20.00) | 2 (4.00) | 0 (0.00) |
| **Scenario #31:** A nurse securely locks the door of the person's room as a safety measure during the daytime. | | | | | |
| Clinicians | 132(63.77) | 37 (17.87) | 22 (10.63) | 15 (7.25) | 1 (0.48) |
| Researchers | 45 (47.87) | 24 (25.53) | 18 (19.15) | 6 (6.38) | 1 (1.06) |
| Service users | 29 (67.44) | 10 (23.26) | 0 (0.00) | 4 (9.30) | 0 (0.00) |
| Family caregivers | 27 (54.00) | 9 (18.00) | 8 (16.00) | 5 (10.00) | 1 (2.00) |
| **Scenario #32:** An individual was kept in a closed, empty room without furniture, windows, light, or toilets to manage agitated behaviour. | | | | | |
| Clinicians | 141(68.12) | 24 (11.59) | 13 (6.28) | 26 (12.56) | 3 (1.45) |
| Researchers | 54 (57.45) | 13 (13.83) | 9 (9.57) | 16 (17.02) | 2 (2.13) |
| Service users | 31 (72.09) | 7 (16.28) | 2 (4.65) | 3 (6.98) | 0 (0.00) |
| Family caregivers | 31 (62.00) | 7 (14.00) | 4 (8.00) | 8 (16.00) | 0 (0.00) |
| **Scenario #33:** An individual is kept in a closed room that is fully furnished with windows, light, and toilets to manage agitated behaviour | | | | | |
| Clinicians | 120(58.54) | 36 (17.56) | 35 (17.07) | 12 (5.85) | 2 (0.98) |
| Researchers | 33 (35.48) | 33 (35.48) | 18 (19.35) | 8 (8.60) | 1 (1.08) |
| Service users | 25 (58.14) | 6 (13.95) | 7 (16.28) | 4 (9.30) | 1 (2.33) |
| Family caregivers | 18 (36.00) | 16 (32.00) | 11 (22.00) | 5 (10.00) | 0 (0.00) |
| **Scenario #34:** A nurse administers sleep-inducing medication to a person suffering from insomnia. Although the hospital has approved the use of this medication, the nurse increases the amount (dosage) of the medication to achieve an immediate clinical response. | | | | | |
| Clinicians | 73 (35.61) | 43 (20.98) | 38 (18.54) | 37 (18.05) | 14 (6.83) |
| Researchers | 27 (29.03) | 22 (23.66) | 24 (25.81) | 16 (17.20) | 4 (4.30) |
| Service users | 19 (44.19) | 11 (25.58) | 5 (11.63) | 7 (16.28) | 1 (2.33) |
| Family caregivers | 17 (34.00) | 17 (34.00) | 4 (8.00) | 11 (22.00) | 1 (2.00) |
| **Scenario #35:** A nurse administers sleep-inducing medication to a person suffering from insomnia**.** This medication is not approved for use in the hospital. However, the nurse decides to administer the medication to achieve an immediate clinical response. | | | | | |
| Clinicians | 94 (46.31) | 16 (7.88) | 32 (15.76) | 49 (24.14) | 12 (5.91) |
| Researchers | 41 (44.09) | 15 (16.13) | 15 (16.13) | 20 (21.51) | 2 (2.15) |
| Service users | 27 (62.79) | 6 (13.95) | 2 (4.65) | 6 (13.95) | 2 (4.65) |
| Family caregivers | 31 (62.00) | 4 (8.00) | 2 (4.00) | 12 (24.00) | 1 (2.00) |
| **Scenario #36:** A nurse administers sleep-inducing medication to a person suffering from insomnia. The hospital has approved the use of this medication, and the nurse administers the prescribed dose according to the hospital’s protocol | | | | | |
| Clinicians | 63 (31.19) | 20 (9.90) | 35 (17.33) | 83 (41.09) | 1 (0.50) |
| Researchers | 29 (31.18) | 9 (9.68) | 21 (22.58) | 30 (32.26) | 4 (4.30) |
| Service users | 11 (25.58) | 2 (4.65) | 17 (39.53) | 12 (27.91) | 1 (2.33) |
| Family caregivers | 12 (24.00) | 12 (24.00) | 14 (28.00) | 10 (20.00) | 2 (4.00) |
| **Scenario #37:** A nurse prevents the person from leaving/exiting a designated area or space | | | | | |
| Clinicians | 90 (44.55) | 67 (33.17) | 26 (12.87) | 17 (8.42) | 2 (0.99) |
| Researchers | 32 (34.41) | 29 (31.18) | 18 (19.35) | 12 (12.90) | 2 (2.15) |
| Service users | 21 (48.84) | 12 (27.91) | 6 (13.95) | 2 (4.65) | 2 (4.65) |
| Family caregivers | 21 (42.00) | 16 (32.00) | 7 (14.00) | 4 (8.00) | 2 (4.00) |
| **Scenario #38:** A nurse prevents the person from receiving visits from family, friends, or loved ones. | | | | | |
| Clinicians | 96 (47.52) | 32 (15.84) | 28 (13.86) | 42 (20.79) | 4 (1.98) |
| Researchers | 35 (37.63) | 22 (23.66) | 13 (13.98) | 21 (22.58) | 2 (2.15) |
| Service users | 25 (58.14) | 7 (16.28) | 6 (13.95) | 3 (6.98) | 2 (4.65) |
| Family caregivers | 28 (56.00) | 7 (14.00) | 3 (6.00) | 12 (24.00) | 0 (0.00) |
| **Scenario #39:** A person is securely locked alone in a room. | | | | | |
| Clinicians | 129(63.86) | 34 (16.83) | 21 (10.40) | 17 (8.42) | 1 (0.50) |
| Researchers | 54 (58.06) | 15 (16.13) | 8 (8.60) | 15 (16.13) | 1 (1.08) |
| Service users | 29 (67.44) | 5 (11.63) | 7 (16.28) | 1 (2.33) | 1 (2.33) |
| Family caregivers | 20 (40.00) | 10 (20.00) | 12 (24.00) | 8 (16.00) | 0 (0.00) |
| **Scenario #40:** A person is securely locked in a room while a staff member is present in the room with the person. | | | | | |
| Clinicians | 91 (45.50) | 38 (19.00) | 38 (19.00) | 25 (12.50) | 8 (4.00) |
| Researchers | 36 (38.71) | 25 (26.88) | 19 (20.43) | 12 (12.90) | 1 (1.08) |
| Service users | 22 (51.16) | 11 (25.58) | 6 (13.95) | 2 (4.65) | 2 (4.65) |
| Family caregivers | 13 (27.08) | 15 (31.25) | 15 (31.25) | 5 (10.42) | 0 (0.00) |
| **Scenario #41:** A person is securely locked in a room together with a group of people | | | | | |
| Clinicians | 91 (45.50) | 38 (19.00) | 38 (19.00) | 25 (12.50) | 8 (4.00) |
| Researchers | 36 (38.71) | 25 (26.88) | 19 (20.43) | 12 (12.90) | 1 (1.08) |
| Service users | 22 (51.16) | 11 (25.58) | 6 (13.95) | 2 (4.65) | 2 (4.65) |
| Family caregivers | 13 (27.08) | 15 (31.25) | 15 (31.25) | 5 (10.42) | 0 (0.00) |
| **Scenario #42:** A nurse applies mechanical restraints to the person without obtaining consent from the person or family members /caregivers. | | | | | |
| Clinicians | 139(69.85) | 26 (13.07) | 11 (5.53) | 22 (11.06) | 1 (0.50) |
| Researchers | 53 (56.99) | 14 (15.05) | 9 (9.68) | 16 (17.20) | 1 (1.08) |
| Service users | 35 (81.40) | 4 (9.30) | 2 (4.65) | 2 (4.65) | 0 (0.00) |
| Family caregivers | 26 (54.17) | 8 (16.67) | 8 (16.67) | 6 (12.50) | 0 (0.00) |
| **Scenario #43:** A nurse threatens the person with mechanical restraint unless the person agrees to take the prescribed medication as directed. | | | | | |
| Clinicians | 93 (46.73) | 47 (23.62) | 33 (16.58) | 25 (12.56) | 1 (0.50) |
| Researchers | 36 (38.71) | 26 (27.96) | 18 (19.35) | 11 (11.83) | 2 (2.15) |
| Service users | 28 (65.12) | 10 (23.26) | 3 (6.98) | 0 (0.00) | 2 (4.65) |
| Family caregivers | 20 (41.67) | 11 (22.92) | 10 (20.83) | 7 (14.58) | 0 (0.00) |
| **Scenario #44:** A nurse forcefully confines a person to a seclusion room based on a report received from the person’s family caregivers**,** stating that the person intends to physically harm others. | | | | | |
| Clinicians | 136(68.34) | 36 (18.09) | 13 (6.53) | 13 (6.53) | 1 (0.50) |
| Researchers | 49 (52.69) | 21 (22.58) | 16 (17.20) | 5 (5.38) | 2 (2.15) |
| Service users | 30 (71.43) | 3 (7.14) | 5 (11.90) | 3 (7.14) | 1 (2.38) |
| Family caregivers | 21 (43.75) | 7 (14.58) | 15 (31.25) | 5 (10.42) | 0 (0.00) |
